# Synergistic In Vitro Antimicrobial Activity of Pomegranate Rind Extract and Zinc (II) against *Micrococcus luteus* under Planktonic and Biofilm Conditions

**DOI:** 10.3390/pharmaceutics13060851

**Published:** 2021-06-08

**Authors:** Vildan Celiksoy, Rachael L. Moses, Alastair J. Sloan, Ryan Moseley, Charles M. Heard

**Affiliations:** 1 School of Pharmacy and Pharmaceutical Sciences, Cardiff University, Cardiff CF10 3NB, UK; CeliksoyV@cardiff.ac.uk; 2 Oral and Biomedical Sciences, School of Dentistry, Cardiff University, Cardiff CF14 4XY, UK; rachael.moses@unimelb.edu.au (R.L.M.); alastair.sloan@unimelb.edu.au (A.J.S.); MoseleyR@cardiff.ac.uk (R.M.); 3 Melbourne Dental School, Faculty Medicine, Dentistry and Health Sciences, University of Melbourne, Melbourne, VIC 3010, Australia

**Keywords:** pomegranate rind extract, zinc (II), synergistic activity, *Micrococcus luteus*, biofilm, pathogens, catheter, polyphenols, punicalagin

## Abstract

Infectious diseases caused by microbial biofilms are a major clinical problem, and new antimicrobial agents that can inhibit biofilm formation and eradicate pre-formed biofilms are urgently needed. Pomegranate extracts are a well-established folkloric medicine and have been used in the treatment of infectious diseases since ancient times, whilst the addition of metal ions, including zinc (II), has enhanced the antimicrobial activity of pomegranate. *Micrococcus luteus* is generally a non-pathogenic skin commensal bacterium, although it can act as an opportunistic pathogen and cause serious infections, particularly involving catheterization and comorbidities. The aims of this study were to evaluate the holistic activity of pomegranate rind extract (PRE), Zn (II), and PRE/Zn (II) individually and in combination against *M. luteus* under both planktonic and biofilm conditions. Antimicrobial activity was detected in vitro using the broth dilution method, and synergistic activity was determined using checkerboard and time-kill assays. Effects on biofilm formation and eradication were determined by crystal violet and BacLight^TM^ Live/Dead staining. PRE and Zn (II) exerted antimicrobial activity against *M. luteus* under both planktonic and biofilm conditions. After 4 h, potent synergistic bactericidal activity was also found when PRE and Zn (II) were co-administered under planktonic conditions (log reductions: PRE 1.83 ± 0.24, Zn (II) 3.4 ± 0.08, and PRE/Zn (II) 6.88 ± 1.02; *p* < 0.0001). In addition, greater heterogeneity was induced in the structure of *M. luteus* biofilm using the PRE/Zn (II) combination compared to when PRE and Zn (II) were applied individually. The activity of PRE and the PRE/Zn (II) combination could offer a novel antimicrobial therapy for the treatment of disease-associated infections caused by *M. luteus* and potentially other bacteria.

## 1. Introduction

Biofilm-related infections are one of the major problems for the economies and health of societies worldwide [1,2]. Biofilms are aggregations of microorganisms, where the cells are embedded within a self-produced matrix or extracellular polymeric substances (EPS) [3]. In addition, microorganisms within a biofilm are less sensitive to antimicrobial agents and antibiotics than when in their planktonic states [4]. *Micrococcus* species can form biofilms on a variety of surfaces, such as human skin, soil, and medical devices [5,6]. While generally accepted as non-pathogenic, *M. luteus* can cause infection as an opportunistic pathogen, especially in immuno-suppressed patients with other comorbidities [7,8,9,10,11,12]. It has been reported that *M. luteus* is associated with septic arthritis, prosthetic valve endocarditis, and recurrent bacteremia [13]. Moreover, while *M. luteus* is considered to be an abundant commensal microbe in healthy skin, it has been found to enhance *S. aureus* pathogenesis [14,15]. In one case report, a patient with native valve endocarditis due to *M. luteus* infection was unsuccessfully treated with vancomycin, gentamicin, and rifampicin [16]. In addition, MDR bacteria represent an enormous global problem in clinical settings, with resistance also being reported with *M. luteus* [17,18]. Hence, while *M. luteus* is currently susceptible to a range of antibiotics, resistance has been reported for ampicillin and erythromycin [19]. There are an increasing number of reports showing that *M. luteus* can cause severe infections such as pneumonia, ventricular shunt-related meningitis, septic arthritis, bacteremia, peritonitis, and endocarditis in immunosuppressive patients [20]. Though typically of low virulence, *M. luteus* may become pathogenic in patients with impaired resistance, such as by colonizing the surface of heart valves [21]. Catheter-related infections are also a challenging medical problem because catheters can be colonized by commensal microorganisms that are found in the skin surrounding the site of catheter insertion [22,23]. *M. luteus* naturally colonizes the skin, mucosae, and oropharynx and has been associated with such infections related to catheterization [10].

**Figure 1:**
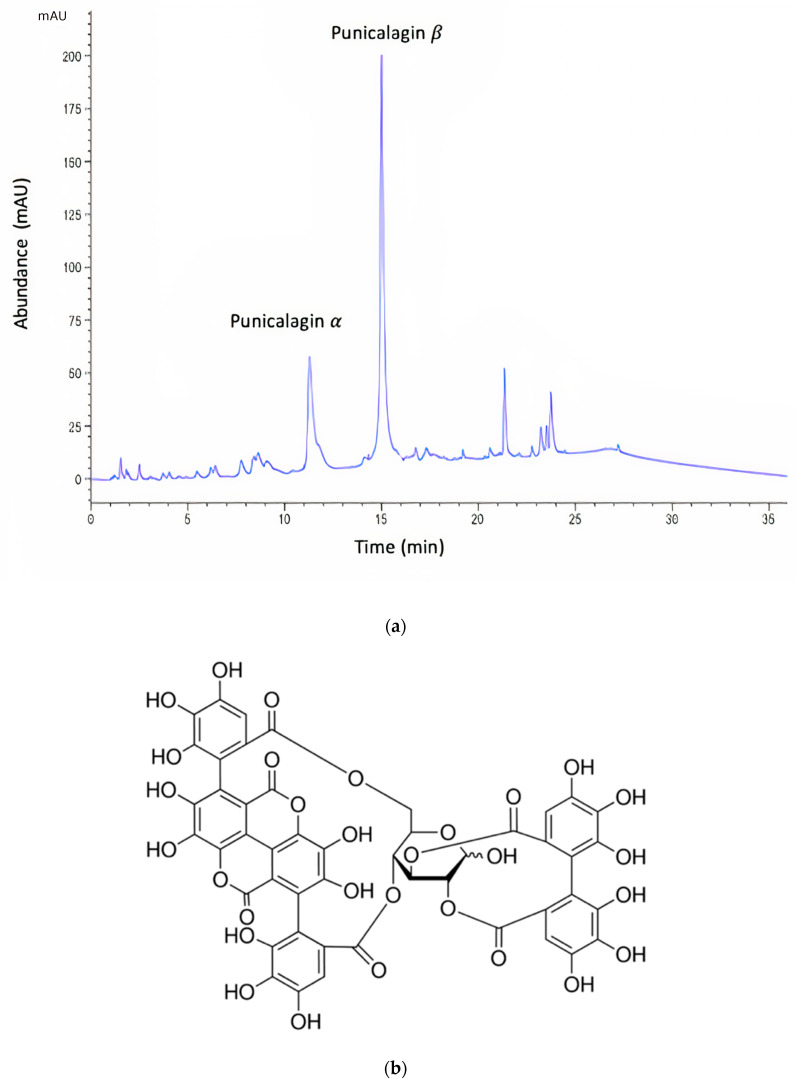
(**a**) Representative HPLC chromatogram of pomegranate rind extract (PRE). Note: the α and β anomers of punicalagin are in a characteristic 1:2 ratio. (**b**) Chemical structure of punicalagin.

Plant extracts continue to be examined as sources of novel antimicrobial agents. Pomegranate (*Punica granatum*) is part of the Punicaceae family native to the Middle East and cultivated in various parts of the world [24]. Pomegranate is a well-established folklore medicine, and it has been used as a traditional medicine for the treatment of dysentery, diarrhea, and stomatitis in many cultures [25]. Recent studies have shown that pomegranate demonstrates benefits in treating numerous conditions due to its anticancer, antimicrobial, anti-inflammatory and antioxidant activities [26]. The pomegranate exocarp, or rind, is abundant in hydrolysable tannins, or ellagitannins, and these compounds have been attributed as being the primary sources of bioactivity responsible for the beneficial medicinal properties of pomegranate [25]. In particular, punicalagin (Figure 1), a large ellagitannin with a molecular weight of 1084.71, has shown antimicrobial activity against a variety of microorganisms [26,27,28].

The combination of antimicrobials with metal ions has offered beneficial novel approaches to treat bacterial infections and in the fight against multi drug resistant (MDR) bacteria [26]. The enhancement of the anti-bacteriophage and antimicrobial activities of pomegranate rind extract (PRE) in combination with metal salts has been reported in several studies, including synergistic (potentiated) virucidal activity against *Herpes simplex* virus I and II [28,29,30,31]. Based on these previous findings, this study evaluated the holistic activity of PRE (rather than its individual chemical constituents), Zn (II), and the combination of PRE/Zn (II) against planktonic bacteria, namely *M. luteus*. We then aimed to determine the effects of these substances against *M. luteus* pre-formed biofilm and biofilm formation.

## 2. Materials and Methods

### 2.1. Materials

Pomegranates (of Spanish origin) were obtained from a local supermarket. Zn (II), as zinc sulfate heptahydrate (ZnSO_4_·7H_2_O), and potassium hydrogen phthalate were obtained from ThermoFisher Scientific (Loughborough, UK). Punicalagin (≥98%), Folin–Ciocalteu (F–C) reagent, ascorbic acid, and sodium carbonate (Na_2_CO_3_) were all obtained from Sigma-Aldrich (Gillingham, UK). Mueller–Hinton broth (MH broth), Mueller–Hinton agar (MH agar), and Brain–Heart Infusion agar (BHIA) were obtained from Oxoid (Basingstoke, UK). The Live/Dead BacLight^TM^ Bacterial Viability Kit was obtained from Invitrogen Molecular Probes (Paisley, UK).

### 2.2. Preparation of Pomegranate Rind Extract (PRE)

Pomegranate rinds were removed using a scalpel and cut to approximately 2 cm^2^ pieces. Then, 300 g of rind were blended (25% *w/v*) in deionized water in a standard blender until visibly homogeneous. This was then boiled for 10 min and centrifuged four times using a Heraeus Multifuge 3S/3S-R centrifuge (5980× *g* at 4 °C for 30 min) before being filtered through 0.45-µm Whatman nylon membrane filter. The filtrate was then freeze-dried, protected from light, and stored at −20 °C until required. The desired concentration of PRE was prepared in a pH 4.5 phthalate buffer and sterilized by using a 0.45 μm Millex-FG syringe-driven filter [32]. PRE and Zn (II) stock solutions were prepared in a phthalate buffer at pH 4.5, then diluted in MH broth to the desired concentrations, and sterilized.

### 2.3. Characterization of PRE

The total phenolic content of PRE was determined using a modified version of the previously described Folin–Ciocalteu colorimetric method [33]. Briefly, 500 mg/L PRE samples were prepared, and 200 µL of 10% (*v/v*) F–C reagent were added to 100 µL of the prepared PRE samples, followed by the addition of 800 µL of 700 mM Na_2_CO_3_. After 2 h of incubation at room temperature, 200 µL of each sample were added to 96-well plates, and the absorbance values were read at 760 nm using a plate reader (Fluostar Optima, BMG Labtech, Aylesbury, UK). The results were expressed as tannic acid equivalent (TAE) per gram of freeze-dried sample. Punicalagin concentrations were determined by HPLC analysis performed with an Agilent 1100 system fitted with a Kinetex C18 150 × 4.6 mm 5 μm 100 Å RP column (Phenomenex, Macclesfield, UK) based on the method reported by Seeram et al. [34].

### 2.4. Microorganism and Test Solutions

*Micrococcus luteus* 59 PIM was used in this study, and its identity was confirmed using a Bruker MALDI Biotyper identification protocol (Bruker Daltonik, Bremen, Germany) (Supplementary Data). Bacteria were grown on either MH agar or BHIA. MH broth was used for overnight cultures, and the stationary-phase inoculum was obtained by inoculating 10 mL of broth for around 20 h at 37 °C under aerobic conditions for each experiment. This was diluted with sterile MH broth to obtain 0.08–0.1 of OD_600_ corresponding to 10^8^ CFU/mL. This OD_600_ range was confirmed by plate count.

PRE and Zn (II) stock solutions were prepared in a pH 4.5 phthalate buffer and then diluted in MH broth (a 10% pH 4.5 phthalate buffer in highest concentrations of working PRE and Zn (II) samples). Only MH broth was added as a sterility control and a 10% phthalate buffer in MH broth was added to observe any possible antimicrobial effect of phthalate buffer pH 4.5 to all microbiology tests. No antimicrobial effect was observed with 10% phthalate buffer in the MH broth. PRE/Zn (II) combination solutions for assays were prepared by combining equal volumes (1:1) of PRE and Zn (II). PRE/Zn (II) (2xMIC + 2xMIC) was prepared by combining same volume of PRE (4xMIC) and Zn (II) (4xMIC) for biofilm inhibition assay as it will be diluted again when it was added on the same volume of bacterial suspension. PRE/Zn (II) (MIC + MIC) was prepared by adding equal volumes of PRE (2 × MIC) and Zn (II) (2 × MIC). Similarly, PRE/Zn (II) (MIC/2 + MIC/2) was prepared by adding equal volumes of PRE (1 × MIC) and Zn (II) (1 × MIC).

### 2.5. Microdilution Method

The minimum inhibitory concentrations (MICs) of the test substances were determined using the broth microdilution method, according to standard guidelines of the CLSI [35]. Overnight cultures of *M. luteus* were prepared in an MH broth until turbid and corresponding to 10^6^ CFU/mL. The serial dilutions of PRE (976–25,000 mg/L) and Zn (II) (2246–28,760 mg/L) were prepared in a 96-well plate using MH broth as a diluent. A 100 µL volume of prepared bacterial suspension was added to each well, which included the same volume of serially diluted compounds. The prepared plates were incubated at 37 °C under aerobic conditions. After 24 h, the plates were also visually evaluated to determine the MIC values as the lowest compound concentrations that inhibited microbial growth. The minimum bactericidal concentration (MBC) was determined according to CLSI guidelines [36]. Briefly, the MBC values for the compounds were determined as the lowest concentration at which no growth was observed in 24 h.

### 2.6. In Vitro Evaluation Synergistic/Antagonistic Activity of PRE and Zn (II)

#### 2.6.1. Checkerboard Assay

The potential for synergism between PRE and Zn (II) was evaluated using a checkerboard assay [37]. The test was performed in MH broth using 96-well microtiter plates containing two-fold serial concentrations of PRE and Zn (II). Then, a bacterial solution was prepared to obtain a final inoculate of 5 × 10^5^ CFU/mL for each well, and plates were incubated for 24 h at 37 °C under aerobic conditions. At the end of the incubation period, the wells were visually examined and the MICs recorded for PRE, Zn (II), and PRE/Zn (II) in combination. Each test was performed in triplicate. The observed MIC values were used to calculate the fractional inhibitory concentration (FIC) of each compound. The FIC value was calculated by dividing the MIC value of the compound in combination with the MIC value of the compound alone. Then, the FIC values of each test substance were added to find the FICindex, as per the following formula:

FIC value of compound A; FICA = (MICA in combination)/(MICA alone)

FIC value of compound B; FICB = (MICB in combination)/(MICB alone)

The FICindex = FICA + FICB FICindex values were interpreted as synergy for FICindex < 0.5, no interaction for 1 < FICindex < 4, or antagonistic for FICindex > 4 [38].

#### 2.6.2. Time-Kill Assay

Time-kill assays against *M. luteus* were performed at four time points (10, 30, 60, and 240 min) for half the MIC (MIC/2) values calculated for PRE, Zn (II), and the PRE/Zn (II) combination. Compounds and their combinations were incubated with 10^6^ CFU/mL of bacteria at 37 °C under aerobic conditions. At each time-point, a sterilized universal quenching agent (0.1% peptone, 0.1% sodium thiosulphate, 0.5% Tween 80, and 0.07% lecithin *w/v* at pH 7) [39] was added to each sample and diluted in sterile phosphate-buffered saline (pH 7.2) for viable bacterial count determination using the Miles and Misra method [40]. Each experiment was repeated twice for three independent experiments, and data are expressed as mean log reduction ± SEM.

### 2.7. In Vitro Biofilm Inhibition and Eradication Activity of PRE, Zn (II), and PRE/Zn (II) Combination

#### 2.7.1. Biofilm Inhibition and Eradication Assay

Biofilm formation was quantified in 96-well plates with standard crystal violet staining using an adaptation of a previously reported biofilm inhibition spectrophotometric assay [41]. Plates were prepared with 100 µL of *M. luteus* suspension (10^8^ CFU/mL), and 100 µL of PRE, Zn (II) and PRE/Zn (II) combinations were added into the wells to obtain MIC and MIC/2 values as final concentrations of compounds and combinations for testing, as described in Section 2.4. In addition, a background plate was prepared for specified concentrations of compounds and combinations without bacterial inoculation. Plates were incubated for 24 h at 37 °C under aerobic conditions. After 24 h, the liquid suspensions were removed and a 100 µL 1% *v/v* aqueous solution of crystal violet was added and incubated for 30 min at room temperature. Then, the dye was removed, the wells washed thoroughly, and 95% ethanol was added and incubated for 20 min. The violet solution was spectrophotometrically determined at 570 nm, as described above. The background optical density (O.D.) value of compounds was subtracted from O.D. treatment and the O.D. control to overcome any non-specific binding of crystal violet. The percent inhibition was then calculated as follows [42]:

% inhibition = 100 − [(O.D. treatment/O.D. control) × 100](1)

For biofilm eradication, a similar method was applied. Briefly, a bacterial inoculate was added to each plate and incubated for 24 h at 37 °C under aerobic conditions on a rotator set at 20 rpm to create the biofilms. After 24 h, fresh test substances and combinations were added using the same conditions as previously described for another 24 h after discarding the supernatants. Next, the same crystal violet method and calculation steps were used to obtain the percentage biofilm eradication.

#### 2.7.2. Fluorescence Microscopy Analysis of Biofilm Eradication

A Live/Dead BacLight^TM^ Bacterial Viability assay was performed to investigate the effects of PRE, Zn (II) and PRE/Zn (II) combination on pre-existing 24 h-old biofilms using glass-bottomed 96-well plates (Greiner Bio One Ltd., Stonehouse, UK). The assay was performed based on the method previously described by Powell et al. [43]. Briefly, a 100 µL aliquot of a bacterial suspension at 10^8^ CFU/mL in MH broth was added into the plate and aerobically incubated for 24 h at 37 °C After 24 h, supernatants were discarded and replaced with PRE (MIC), Zn (II) (MIC), and PRE/Zn (II) combination (MIC + MIC) solutions and incubated at 37 °C for 24 h. Then, each plate was stained using the Live/Dead BacLight^TM^ Bacterial Viability Kit according to manufacturer’s instructions and visualized using the Leica TCS SP5 Confocal Microscope (Leica Microsystems Ltd., Milton Keynes, UK). Images were obtained with a 60 × 1.8 oil objective and a z-step size of 1 µm. Z-stack images obtained using Imaris software (Bitplane, Concord, MA, USA) were analyzed by using Comstat2 plugin with the ImageJ analysis software, Version 2.1.0 (U. S. National Institutes of Health, Bethesda, Maryland, USA) [44] and results are expressed as mean ± SEM (*n* = 12).

### 2.8. Statistical Analysis

Experiments were performed in triplicate by using independent microbial cultures for all antimicrobial assays. The results were analyzed and graphically presented with the GraphPad Prism software (GraphPad Software, Version 8.2.1, San Diego, CA, USA). A one-way ANOVA test with Tukey correction was used, and *p* < 0.05 was considered statistically significant.

## 3. Results

### 3.1. Characterization of PRE

The Folin–Ciocalteu (F–C) assay showed that freeze-dried PRE contained an average of 496 mg of TAE/g. The PRE extract was analyzed by reverse-phase HPLC [32], which showed two characteristic major peaks corresponding to the α and β anomers of punicalagin, which are known to interconvert spontaneously to a ratio of punicalagin β to punicalagin α content in the range of 2:1–1.6:1 [32,34,45]. In the current study, the ratio of punicalagin β to punicalagin α content was found to be 1.76:1 (Figure 1). Using pure punicalagin as a standard, it was determined that the amount of total punicalagin in a 1000 mg/L aqueous solution of PRE was 170 µg [32].

### 3.2. Determination of MIC and MBC Values of PRE and Zn (II)

The MIC values of PRE and Zn (II) against *M. luteus* are shown in Table 1. Inhibitory activity was found at 1560 and 1790 mg/L for PRE and Zn (II), respectively. As expected, the bactericidal concentrations of both compounds were higher than their inhibitory counterparts. Thus, it could be suggested that each compound’s antimicrobial activities are based on their inhibitory activities, rather than bactericidal activities.

### 3.3. Determination of Synergistic Activity of PRE/Zn (II) in Combination

**Figure 2:**
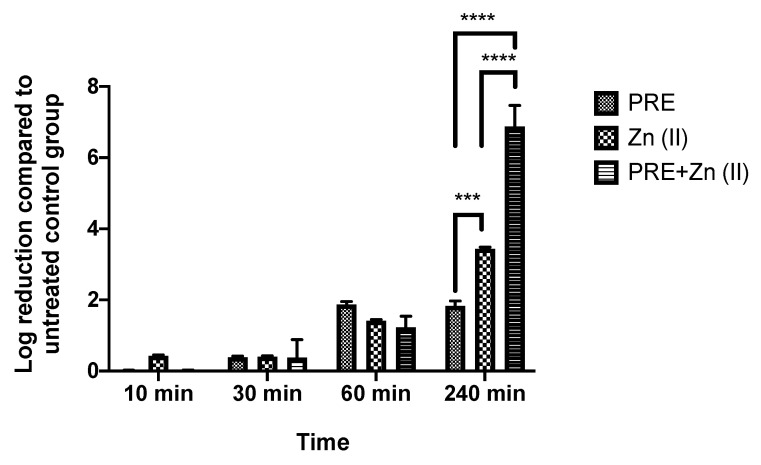
Log-reduction values obtained using time-kill assays for PRE (MIC/2), Zn (II) (MIC/2), and PRE/Zn (II) (MIC/2 + MIC/2) in combination against *M. luteus*. All data are represented as mean ± SEM (*n* = 3). Note: synergistic activity at 240 min, showing a 6.88 ± 1.02 log reduction for PRE/Zn (II), which was significantly greater than the 1.83 ± 0.24 log reduction for PRE and the 3.4 ± 0.08 log reduction for Zn (II); statistical significance indicated at *** *p* < 0.001 and **** *p* < 0.0001 between treatment groups.

Both PRE and Zn (II) showed activity against *M. luteus* under planktonic conditions, and the possibility of synergistic antimicrobial activity of PRE and Zn (II) in combination was assessed using in vitro checkerboard and time-kill kinetic assays. The obtained FICindex value was <0.5 (Table 2), and it was accepted as a demonstration of the synergistic activity of the combination. At 240 min significant synergistic activity was observed in the time-kill assay (Figure 2), where the PRE/Zn (II) combination gave a log reduction of 6.88 ± 1.02 compared to 1.83 ± 0.24 for PRE alone and 3.4 ± 0.08 for Zn (II) alone (*p* < 0.0001). Such synergy was not observed up to 1 h.

### 3.4. Biofilm Inhibition and Eradication Activity of PRE, Zn (II) and PRE/Zn (II) in Combination

#### 3.4.1. Percentage of Biofilm Inhibition and Eradication through In Vitro Crystal Violet Assay

**Figure 3:**
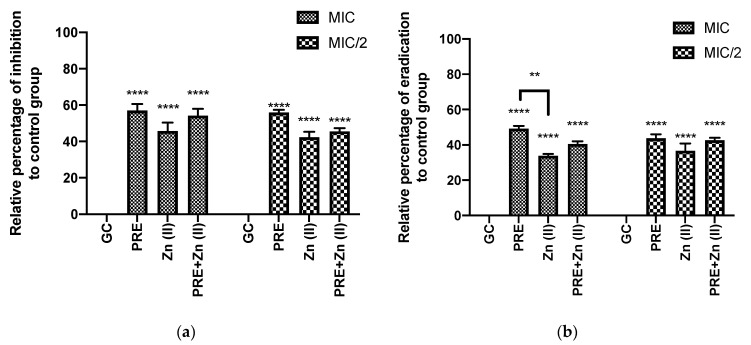
(**a**) Percentage biofilm inhibition and (**b**) biofilm eradication by PRE, Zn (II) and PRE/Zn (II) in combination at MIC and half of MIC values (MIC/2) against *M. luteus*, using the in vitro crystal violet assay. All data are represented as mean ± SEM, *n* = 3. Statistical significance indicated at ** *p* < 0.01, and **** *p* < 0.0001, compared to untreated growth controls and compared between treatment groups. Note: a significant effect of PRE, Zn (II), and the PRE/Zn (II) combination in reducing biomass compared to untreated growth control group in biofilm inhibition and biofilm eradication activities.

Figure 3 shows that PRE (MIC/2, MIC), Zn (II) (MIC/2, MIC), and PRE/Zn (II) in combination (MIC + MIC and MIC/2 + MIC/2) each exhibited biofilm inhibition and eradication when using in vitro crystal violet assay. In this study, the test substances and their combination exerted significant inhibition and eradication activity compared to the untreated control group (*p* < 0.0001). However, synergistic activity was not observed between PRE and Zn (II), as no significant difference was observed between the PRE/Zn (II) combination and the compounds alone (*p* > 0.05).

#### 3.4.2. Fluorescence Microscopy Analysis of Biofilm Eradication

**Figure 4:**
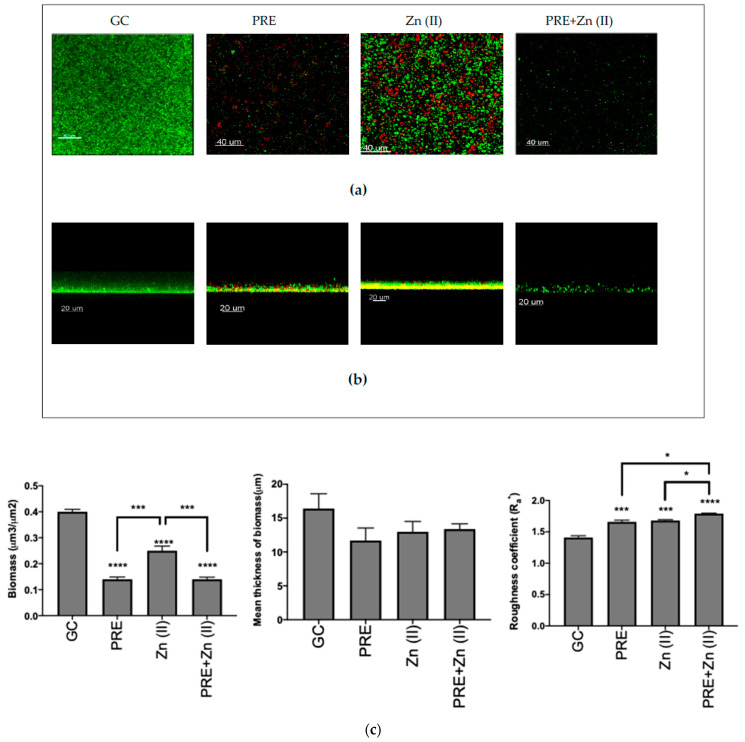
Biofilm eradication assay of *M. luteus* showing Live/Dead (green/red, respectively) stained confocal laser scanning microscopy (CLSM) images in 3D (**a**) and 2D (**b**) views. Scale bars = 40 and 20 µm, respectively. PRE (MIC), Zn (II) (MIC), and PRE/Zn (II) (MIC+MIC) were used. (**c**) Corresponding COMSTAT analyses of the images are shown. Mean ± SEM; n = 12. Statistical significance indicated at * *p* < 0.05, *** *p* < 0.001, and **** *p* < 0.0001, compared to untreated controls and compared between treatment groups. Note: 24-h-old *M. luteus* biofilm disruption following treatment with PRE, Zn (II), and the PRE/Zn (II) combination, as indicated by a decreased biomass and an increased roughness coefficient. The PRE/Zn (II) combination caused greater biofilm destruction compared to both the PRE and Zn (II) combination and the untreated growth control group.

The eradication activity of (MIC), Zn (II) (MIC), and PRE/Zn (II) in combination (MIC + MIC) against the pre-formed *M. luteus* biofilm was examined using CLSM (Figure 4a,b), and quantitative analysis was performed via COMSTAT analysis (Figure 4c). In the control, *M. luteus* formed a thick and compact biofilm when not exposed to any test substance challenge. However, all compounds and the combination significantly decreased the biomass of the biofilm compared to untreated controls, thus disrupting the biofilm (all *p* < 0.0001). In particular, PRE and the PRE/Zn (II) combination caused a significantly greater reduction in biomass than Zn (II) alone (*p* < 0.001). In addition, PRE and Zn (II) increased the roughness coefficient, a parameter which is indicative of biofilm heterogeneity. The combination of PRE and Zn (II) caused a statistically significant increase in the roughness coefficient than both compounds alone (*p* < 0.05). However, no significant differences were observed in the mean thickness values between substances and their combination compared to untreated controls (*p* > 0.05).

## 4. Discussion

The antimicrobial effects of pomegranate have been extensively studied, and different parts of the pomegranate show antimicrobial activity against a range of Gram-positive and Gram-negative bacteria [46,47,48,49]. The pomegranate rind has more polyphenol content than other parts of the fruit and shows more antimicrobial activity than the seeds, whole fruit, and juice extracts [50]. Melendez and Caprilles reported that pomegranate fruit extracts have a marked in vitro antimicrobial activity against a range of bacteria, including *M. luteus* [49]. Negi and Jayaprakasha studied the antimicrobial activity of pomegranate peel in different solvent extracts (acetone, methanol, and water) and found that all extracts exhibited antimicrobial activity against both Gram-positive and Gram-negative bacteria [51]. Duman et al. studied aril extracts from six pomegranate cultivars and found antimicrobial activity against *M. luteus* in all of them [52]. In accordance with the present results, previous studies have demonstrated that the MIC of a pomegranate peel extract was 3130 mg/L against *M. luteus* [53]. In this study, growth inhibition was observed for both PRE and Zn (II) versus *M. luteus*, and the MIC was found to be similar at 1560 mg/L for PRE. 

The current study has shown for the first time that PRE and Zn (II) results in synergistic antibacterial (checkerboard assay) and bactericidal (time-kill assay) activities against *M. luteus*, although the latter only after a prolonged exposure time of 4 h. The reason for this timing is unclear, although the combination resulted in a highly significant doubling of the log reduction. Synergistic virucidal activity between PRE and Zn (II) against *Herpes simplex* virus was previously established with a time-kill assay by Houston et al. [28,32]. The PRE and zinc sulfate combination showed antimicrobial activity against *B. subtilis*, *Staphylococcus* spp., and *Brucella* spp. in zone of inhibition assays [29], although the interpretation of such analyses of complex mixtures and combined compounds in zone inhibition assay is typically difficult due to the different diffusion rates of individual compounds and combinations through the agar. McCarrell et al. studied the antimicrobial activity of PRE with a range of metal salts against Gram-positive and Gram-negative bacteria and found that PRE in combination with zinc sulfate provided no evidence of synergistic activity [29]; they also reported that PRE did not show any antimicrobial activity when used alone. However, they did observe an enhanced antimicrobial activity against *P. aeruginosa*, *P. mirabilis*, and *E. coli* when PRE was combined with the (more toxic) copper (II) ions within 30 min of exposure in a time-kill assay. The antimicrobial activity of PRE is highly associated with its polyphenolic content, and punicalagin is by a long way the most abundant of all compounds in PRE. It was suggested that gallagyl and galloyl-hexahydroxydiphenoyl (HHDP) moieties of compounds in PRE have roles in antimicrobial activity. Recent studies have reported the antimicrobial and antibiofilm activities of punicalagin against *S. aureus* and other microbes [54,55]. It is possible the activity could also involve the synergistic activity of polyphenolic compounds in PRE and its combination with Zn (II) rather than just punicalagin. However, Houston et al. [28] found that PRE and punicalagin performed similarly on a ‘mass-to-mass’ basis against HSV. Differences between MICs and the enhanced antimicrobial activity of PRE/Zn (II) could be caused by a range of factors including different bacterial strains, the harvesting time of fruits, extraction methods, type of metal salts, and type of solvents [56]. This variability could be circumvented using a standardized extraction method from same cultivars of pomegranate fruit, and further analyses are needed to probe potential structure–activity relationships between Zn (II) and other secondary compounds in PRE.

Microbes behave differently and are generally more resistant against antimicrobial agents and antibiotics under biofilm conditions than in their planktonic states [57,58]. It has been hypothesized that *M. luteus* colonizes skin wounds by using the underlying basement membrane substratum, which provides a foundation for the formation of a mature biofilm that makes the environment more suitable for other, more pathogenic bacteria, such as *P. aeruginosa* and *S. aureus*, which are predominant microbes in chronic skin wounds [59,60]. Thus, by inhibiting biofilm formation and eradicating the pre-formed biofilm of *M. luteus*, the generation of more pathogenic biofilms could be prevented [61,62]. 

Antimicrobial and antibiofilm activities PRE and zinc have been reported against a wide range of microbes. Zinc is an essential mineral that plays a vital role in the cellular functions of life organisms, including bacteria; however, it can be toxic to bacteria in higher concentrations, and the broad antimicrobial activity of zinc has been reported by inhibiting the growth of bacteria and interfering with bacterial conjugation [63,64,65,66]. The antibiofilm activity of zinc have been reported against different Gram-positive and Gram-negative microbes including *S. aureus*, *S. mutans*, and *P. aeruginosa* [67,68]. Several reports have shown the anti-biofilm activity of pomegranate peel extracts. In an in vitro study, pomegranate peel alcoholic extracts were assessed on bacteria collected from patients with dental caries or periodontal disease, ultimately inhibiting a range of bacteria in both planktonic and biofilm conditions [69]. In addition, PRE was shown to inhibit the formation of biofilms of *Staphylococcus aureus*, MRSA, *Escherichia coli*, and *Candida albicans*, as well as to disrupt the pre-formed biofilm of *Candida albicans* [70]. Punicalagin’s antimicrobial and antibiofilm activities against *S. aureus* biofilm formation was also demonstrated [55].

Specifically, PRE, Zn (II), and PRE/Zn (II) in combination exerted both biofilm inhibition and biofilm eradication activities, as confirmed by both spectrophotometric assays and Live/Dead staining with CLSM. PRE and PRE/Zn (II) in combination also caused greater decreases in biofilm biomass than Zn (II) alone. This may be explained by the fact that the inhibitory activity on biofilm formation for combined PRE/Zn (II) could be attributed to the activity of PRE rather than Zn (II). However, it should be noted that PRE/Zn (II) in combination significantly affected the roughness coefficient when compared to PRE or Zn (II) alone.

A roughness coefficient provides information about the variability of thickness of the biofilm, is a sign of biofilm heterogeneity [44], and is a commonly used criterion to describe a biofilm’s structure when comparing biofilms [71,72,73]. While PRE, Zn (II), and the PRE/Zn (II) combination reduced the mean thickness of biomass, they did not cause any significant differences compared to control group, possibly because the mean thickness is a 3-dimensional aspect of the biofilm [41] and the compounds and combination in this study caused areal eradication in biofilm that could be observed from the 3D and 2D images, as shown in Figure 4a,b.

*M. luteus* was found and associated with the central venous catheter infection [13,74,75]. Reducing the colonization insertion hub, the site of the catheter, the inhibition of adhesion, and the growth of pathogens that reach the catheter have been suggested as successful preventive strategies for catheter-related infectious diseases [76]. In this study, the PRE/Zn (II) combination exerted potentiated activity in reducing colonization and inhibiting growth control. The anti-adhesive activity of PRE has been confirmed in the literature [77,78]. PRE’s activity on biofilm formation, inhibition and eradication could support the potential efficacy of PRE and the PRE/Zn (II) combination as a preventive agent in catheter-related infectious diseases caused by *M. luteus*, e.g., as a coating on the catheter surface prior to use.

The antimicrobial activity of PRE against *M. luteus* has been shown in previous studies [47,51], but PRE/Zn (II) combinations also gave synergistic antimicrobial activity in planktonic condition against *M. luteus* showed in this study. Moreover, to the best of our knowledge, our study is the first to demonstrate the anti-biofilm properties of PRE and the PRE/Zn (II) combination on biofilms. The antimicrobial mechanism of action for PRE and PRE/Zn (II) is still to be confirmed, although there have been reports that suggested that PRE induces protein precipitation and enzyme inactivation [47,79]. With such protein precipitation and inhibition activities, PRE could inhibit proteins involved in biofilm formation, such as adhesins [80]. This may explain the reduced biofilm formation capabilities of *M. luteus* following treatment with PRE and PRE/Zn (II). Furthermore, it has been shown that tannins are capable of modifying the surface charge of proteins, which helps prevent cell–substratum interactions and biofilm formation [47,79,80]. In general, the antimicrobial activity of PRE has been associated with pomegranate ellagitannins, especially punicalagin (its major tannin constituent) and ellagic acid [48,79,81]. 

Punicalagin and PRE have been shown to downregulate inflammatory mediators [27,82], and enhanced activity between PRE and Zn (II) was reported in in vitro wound healing experiments [83]. However, the mechanism involved the synergistic antimicrobial effect of PRE/Zn (II) in combination is currently unclear. One suggested mechanism is that pomegranate tannins form ‘complexes’ with metallic ions that may exhibit enhanced toxicity towards microbes [84]. Alternatively, the enhanced activity may be independent of complex formation, with components of PRE, e.g., punicalagin and Zn (II), acting independently against different aspects of the bacteria life processes [85,86]. 

## 5. Conclusions

Both PRE and Zn (II) showed antimicrobial activity against *M. luteus* under planktonic and biofilm conditions. PRE/Zn (II) in combination only showed synergistic microbicidal and antimicrobial activity under planktonic conditions. Though the PRE/Zn (II) combination did not show an enhanced antimicrobial activity in the inhibition of biofilm formation, it did significantly alter biofilm physical characteristics by increasing the roughness coefficient compared to biofilm treated with PRE or Zn (II) alone. Overall, the antimicrobial activity of PRE and PRE/Zn (II) against biofilm formation and pre-formed biofilms could represent a promising new treatment for disease-associated infections caused by *M. luteus.*

## Figures and Tables

**Table 1:** Minimum inhibitory concentrations (MICs) and minimum bactericidal concentrations (MBCs) values of PRE (mg/L) and Zn (II) (mg/L) against *M. luteus* in broth suspensions. Presented data are the result of three independent experiments.

Compounds	MIC (mg/L)	MBC (mg/L)
PRE	1560	>1790
Zn (II)	440	>1790

**Table 2:** FICindex values determined for PRE and Zn (II) alone and in combination using an in vitro checkerboard assay against *M. luteus*.

	FICI (PRE)	FICI (Zn (II))	FICI	Conclusion
*M. luteus*	0.0625	0.0625	0.125	Synergy

## Data Availability

The data presented in this study are available on request from the corresponding author.
